# A Promising Material for Biomedicine and Food Production Based on a Polymethyl Methacrylate-like Resin with Silicon Dioxide Nanoparticles

**DOI:** 10.3390/molecules30244740

**Published:** 2025-12-11

**Authors:** Fatikh M. Yanbaev, Dmitriy N. Ignatenko, Ilya V. Baimler, Lev R. Sizov, Dmitriy A. Serov, Alexander V. Simakin, Ruslan M. Sarimov, Valeriy A. Kozlov, Vladislav S. Gudkov, Maksim Rebezov, Alexander D. Kurilov, Mikhail V. Dubinin, Konstantin V. Sergienko, Mikhail A. Sevostyanov, Maxim E. Astashev, Sergey V. Gudkov

**Affiliations:** 1 Federal Research Center “Kazan Scientific Center of the Russian Academy of Sciences”, ul. Lobachevskogo 2/31, 420088 Kazan, Tatarstan, Russia; aurum_fr@mail.ru (F.M.Y.); dmitriyek13104@yandex.ru (D.N.I.); 2 Prokhorov General Physics Institute of the Russian Academy of Sciences, Vavilov Str. 38, 119991 Moscow, Russia; ilyabaymler@yandex.ru (I.V.B.); leo.sizoff@yandex.ru (L.R.S.); dmitriy_serov_91@mail.ru (D.A.S.); avsimakin@gmail.com (A.V.S.); rusa@kapella.gpi.ru (R.M.S.); ad.kurilov@gmail.com (A.D.K.); dubinin1989@gmail.com (M.V.D.); astashev@yandex.ru (M.E.A.); s_makariy@rambler.ru (S.V.G.); 3 Department of Fundamental Sciences, Bauman Moscow State Technical University, 2nd Baumanskaya St. 5, 105005 Moscow, Russia; gudkovvs2006@gmail.com; 4 Department of Scientific Research, Gorbatov Research Center for Food Systems, Talalikhin Str. 26, 109316 Moscow, Russia; rebezov@ya.ru; 5 Faculty of Biotechnology and Food Engineering, Ural State Agrarian University, Karl Liebknecht Str. 42, 620075 Yekaterinburg, Russia; 6 Educational and Scientific Laboratory of Theoretical and Applied Nanotechnology, Federal State University of Education, Radio St. 10a, 105005 Moscow, Russia; 7 Department of Biochemistry, Cell Biology and Microbiology, Mari State University, pl. Lenina 1, 424001 Yoshkar-Ola, Russia; 8 Baikov Institute of Metallurgy and Materials Science, Russian Academy of Sciences, 119334 Moscow, Russia; shulf@yandex.ru (K.V.S.); cmakp@mail.ru (M.A.S.)

**Keywords:** polymethyl methacrylate-like resin, MSLA, silicon dioxide nanoparticles, polymer composite, antibacterial and non-toxic to mammalian cells

## Abstract

Silicon dioxide (SiO_2_) nanoparticles approximately 5 nm in size have been obtained. A method has been developed for introducing SiO_2_ nanoparticles into photolithographic resin at concentrations up to 0.1%. Composite resins can be used to manufacture parts with complex geometries with a maximum achievable resolution of 50 μm. Parts made from composite resin with SiO_2_ nanoparticles polish well. After polishing, areas of approximately 100 μm^2^ with height differences of less than 10 nm are revealed on the surface of the parts. A relatively uniform distribution of SiO_2_ nanoparticles is observed within the parts, and no optical defects are detected. However, areas differing in the phase shift of electromagnetic radiation are observed within the parts. Importantly, the presence of nanoparticles in the resin during MSLA printing increases the degree of resin polymerization. SiO_2_ nanoparticles have been shown to have prooxidant properties, leading to the formation of 8-oxoguanine in DNA and long-lived reactive protein species. Components made from photolithographic resins with SiO_2_ nanoparticles have been shown to inhibit the growth and development of *E. coli* bacteria, with a significant loss of viability. Despite their antimicrobial properties, components made from photolithographic resins with SiO_2_ nanoparticles do not affect the growth and development of mammalian cells.

## 1. Introduction

Achievements of nanotechnology in polymer science today find applications ranging from the food industry to advanced medical technologies [1]. A primary application of nanotechnology in polymer science is the development of nanocomposites based on polymers containing nanoscale objects [2]. The primary advantage and basis for the high activity of nanoscale objects is their enormous surface area-to-mass ratio [3]. The presence of nanoparticles in a polymer often imparts new mechanical, thermal, chemical, and biological properties to products and components made from such polymer [4]. Among all the biological properties, the most interesting are the antimicrobial properties of nanocomposites [5]. Polymer products with antimicrobial properties are in high demand in biomedical applications [6], as well as in the food industry [7] for components such as conveyor parts, seals, process line elements, and food packaging materials [8].

Inorganic nanoparticles are most commonly used in components and products for bionanotechnology applications [9]. Examples include CaO nanoparticles in regenerative medicine products [10], TiO_2_ nanoparticles in photodegradation applications [11], and ZnO nanoparticles among others as antimicrobial additives [12]. Silicon dioxide (silica) nanoparticles combine all the aforementioned capabilities: they can be used in regenerative medicine [13], are suitable for photodegradation [14], and possess significant antimicrobial potential [15]. Silicon dioxide (SiO_2_) is transparent and colorless. It is also insoluble in water, meaning it will not release substances when introduced into the body. It exhibits high hardness and strength, enabling it to maintain its properties and qualities over extended periods [16]. Humanity has been in contact with SiO_2_ since its inception, as it is primary component of sand, making it a relatively safe compound for the body [17]. Today, SiO_2_ is used in the food industry as an anticaking agent (E551) to prevent clumping [18], is a component of toothpastes [19], and serves as a thickener and excipient in the pharmaceutical industry [20].

Currently, final polymer products and components are often still produced by molding or casting [21]. However, additive technologies are gaining popularity [22]. These technologies allow for specific thickness at each point or node of a product, ultimately enabling the fabrication of fully optimized structures with minimal material waste [23]. This applies potentially to a wide range of products, from medical catheters to food packaging. Additive technologies can also create products and components with varying properties at different points or nodes [24]. A prime example is the need for a gradient in mechanical properties along propeller blades, from the hub to the tip [25]. Often, antimicrobial properties are needed only at the surface, not throughout the entire volume of the product or component [26].

While significant portion of known polymers are used in additive manufacturing, the spectrum of suitable polymers narrows considerably when discussing applications in the food industry and biomedicine [27]. High transparency is often required for biomedical and food industry applications [28], meaning the polymer must interact weakly with visible light radiation. For products used in electrical appliances or near power sources, dielectric properties are essential [29]. High resistance, both mechanical and chemical, is crucial [30]. Polymethylmethacrylate (PMMA)-like polymers possess most of these advantages [31]. They are transparent to electromagnetic radiation across a broad spectrum [32], act as dielectrics [33], and are mechanically [34] and chemically [35] resistant. It is important to note that PMMA polymers are biocompatible but lack inherent antimicrobial properties [36]. Thus, a pressing scientific and technical challenge arises, which motivates this study: the existing potential of additive technologies for creating products with complex functional distribution cannot be fully realized for critical applications due to the lack of suitable functional materials.

The current state of research demonstrates significant interest in nanocomposites based on PMMA and SiO_2_ nanoparticles. However, their systematic study specifically in the context of compatibility with additive technologies, particularly photopolymer 3D printing, and the subsequent comprehensive assessment of both the technological (the effect of nanoparticles on the polymerization process and product quality), functional (mechanical and optical properties), and, most importantly, biological (antimicrobial efficacy and cytocompatibility) characteristics of the resulting products remain insufficiently studied. Filling this gap is a necessary step in moving from laboratory development to the creation of finished, safe, and functional products, such as customized medical components or food industry equipment parts manufactured using 3D printing.

Therefore, the aim of this work is to fabricate products and components from a composite photolithographic resin based on PMMA with SiO_2_ nanoparticles that are non-toxic to mammalian cells and exhibit significant antimicrobial properties.

## 2. Results

**Figure 1:**
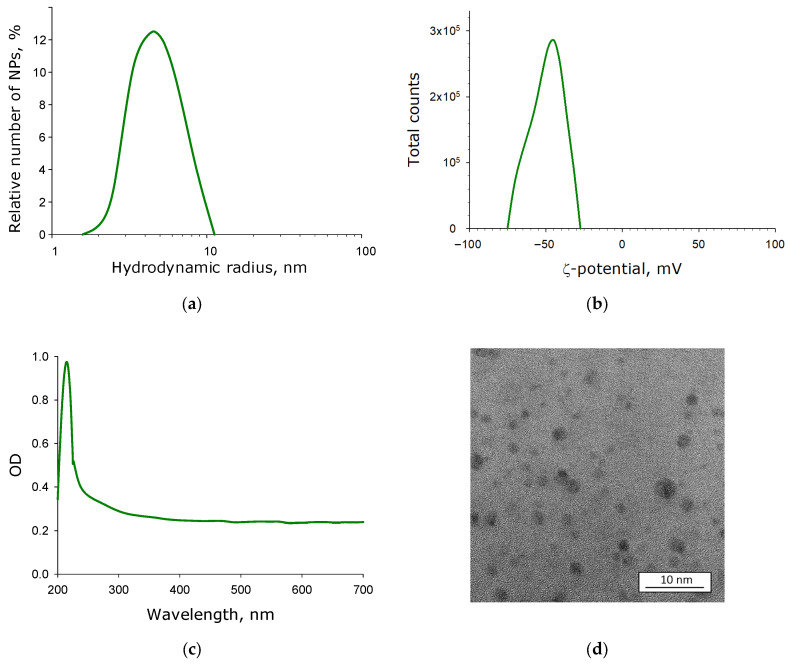
Characteristics of the physicochemical properties of the SiO_2_ nanoparticle preparation obtained by laser ablation. Size distribution of SiO_2_ nanoparticles in aqueous colloid (**a**). ζ-potential distribution of SiO_2_ nanoparticles in aqueous colloid (**b**). Absorption spectrum of the SiO_2_ nanoparticle colloid in the UV and visible ranges (**c**). Transmission electron microscopy micrograph of SiO_2_ nanoparticles (scale bar 10 nm) (**d**).

The size distribution of SiO_2_ nanoparticles was investigated by dynamic light scattering (Figure 1a). The size distribution was shown to be monomodal (exhibiting a single maximum). The average hydrodynamic radius of the SiO_2_ nanoparticles was approximately 2–3 nm. The colloid contained nanoparticles with hydrodynamic radii ranging from 1 to 5 nm. The full width at half maximum of the distribution corresponded to a hydrodynamic radius range of 1.5–3.5 nm.

The ζ-potential distribution of SiO_2_ nanoparticles in the colloid was investigated by dynamic light scattering (Figure 1b). The distribution was monomodal (displaying single maximum). The average ζ-potential of the SiO_2_ nanoparticles was approximately −40 mV. The colloid contained nanoparticles with ζ-potential values ranging from −25 to −75 mV. The full width at half maximum of the distribution corresponded to a ζ-potential range from approximately −30 to −60 mV.

The optical properties of the SiO_2_ nanoparticle colloid in the visible and ultraviolet ranges were studied using a double-beam differential spectrophotometer (Figure 1c). The absorption spectrum of the aqueous colloid showed a distinct maximum at approximately 230 nm. An exponential decrease in optical density was observed in the 230–400 nm wavelength range.

The morphology of the SiO_2_ nanoparticles was examined by transmission electron microscopy (Figure 1d). SiO_2_ exhibits relatively weak contrast. The nanoparticles were predominantly spherical, with an average size ranging from 1 to 5 nm.

**Figure 2:**
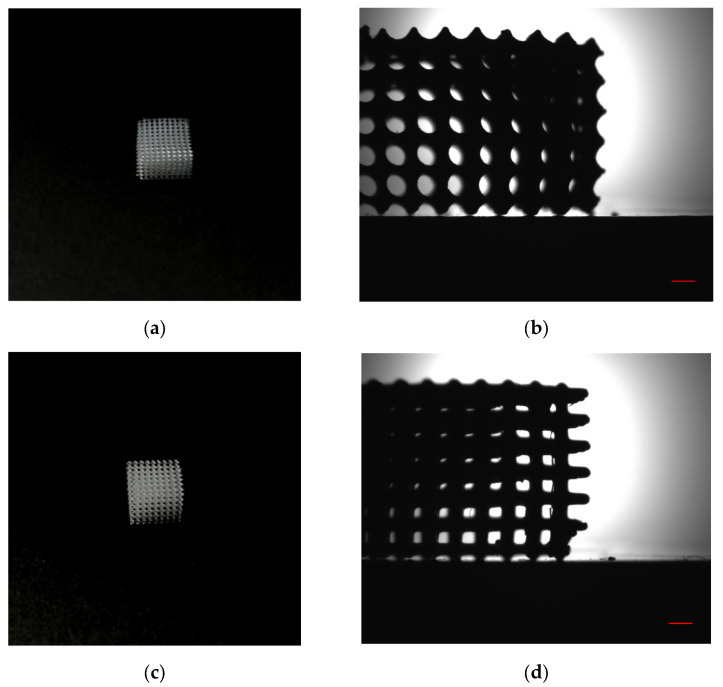
Images of scaffold samples fabricated via masked stereolithography (MSLA) technology from lithographic resin containing 0.1% SiO_2_ nanoparticles. Scaffold with cylindrical channels: macroscopic photograph (**a**) and shadow micrograph (**b**). Scaffold with rectangular channels: macroscopic photograph (**c**) and shadow micrograph (**d**). Scale bar in the shadow micrographs: 1 mm.

SiO_2_ nanoparticles were synthesized by laser ablation. It is known that lithographic resins are insoluble in water, and water is insoluble in lithographic resins. The manufacturing of the scaffold samples shown in Figure 2 from lithographic PMMA-like resin with SiO_2_ nanoparticles required solving the challenge of complete water removal from the resin.

The SiO_2_ nanoparticles were transferred from water to pure acetone. This was achieved by separating the nanoparticles from water via centrifugation (20 h, 21.984× *g*). The water was decanted and replaced with pure acetone. The nanoparticles were resuspended from the bottom of the centrifuge tube into the liquid using an ultrasonic bath (10 min). After this procedure, trace amounts of water (less than 0.1% by volume) remained in the acetone colloid. Adding such colloid to the lithographic resin allowed for object fabrication, but the resulting samples often contained defects. When the solvent exchange procedure was repeated a second time with pure acetone, the quality of the lithographic resin improved. Defects in printed objects were still present but rare. After performing the solvent exchange a third time, no water remained in the colloid. Mixing this anhydrous colloid with the lithographic resin enabled the printing of defect-free objects.

These specific lithographic resins, containing SiO_2_ nanoparticles at concentrations of 0.001% to 0.1%, were used in the sample fabrication process. The printing technology allows for the manufacture of complex-shaped, optically transparent parts from resins containing SiO_2_ nanoparticles. Scaffolds with cylindrical (Figure 2a) and rectangular (Figure 2c) channels exhibited precise geometric forms at both the channel and macro levels. In scaffolds with rectangular channels, the formation of thin filaments within the channel body was extremely rare (fewer than 1 in 1000 instances). One such image is presented here (Figure 2d). In contrast, such artifacts were not observed in scaffolds with cylindrical channels (Figure 2b). Overall, the addition of SiO_2_ nanoparticles to the resin did not significantly affect the spatial resolution. The maximum resolution achieved matched that of the masked stereolithography (MSLA) printer and was approximately 0.05 mm. In addition to scaffolds, thin, round, optically transparent plates were fabricated and used in most subsequent studies.

**Figure 3:**
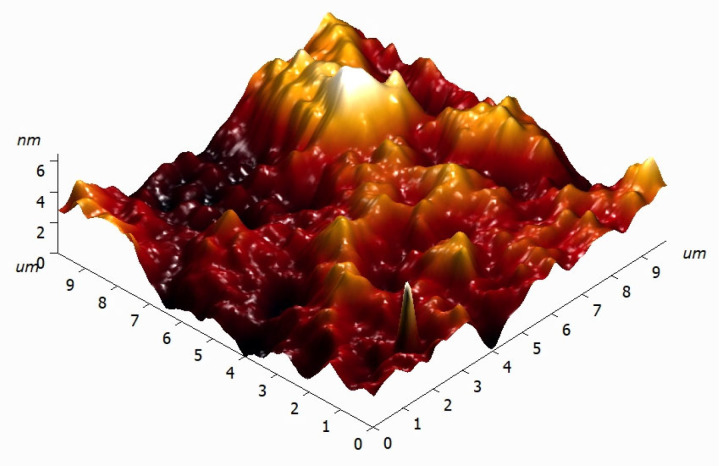
3D surface reconstruction of plate fabricated from lithographic resin containing 0.1% SiO_2_ nanoparticles.

Various samples with extended planar surfaces were fabricated from lithographic resins with and without SiO_2_ nanoparticles. These surfaces were ground and subsequently polished. Polishing was performed until the surfaces produced clear, undistorted reflections of objects in visible light with sharp boundaries. The polished surfaces of the components were examined using atomic force microscopy. Typical three-dimensional surface profile of plate fabricated from photolithographic resin containing 0.1% SiO_2_ nanoparticles is shown in Figure 3. It was demonstrated that within a 10 × 10 μm area of interest, the maximum height variation is on the order of several nanometers.

**Figure 4:**
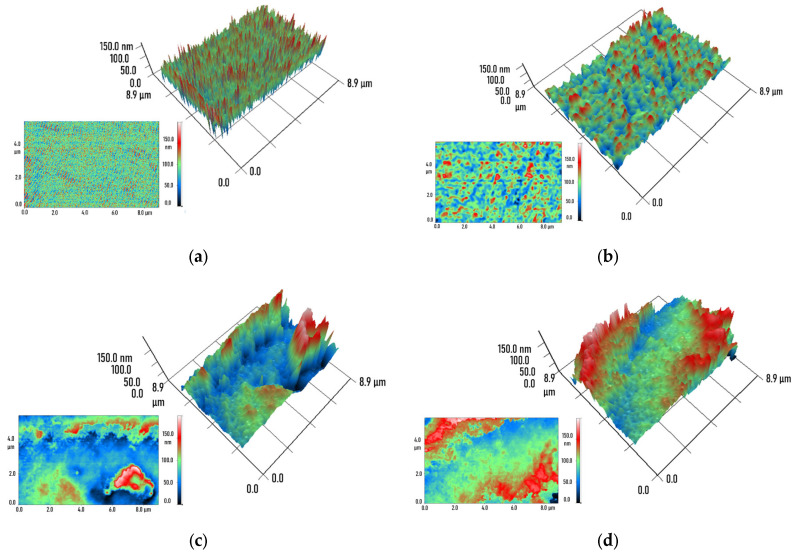
3D reconstruction of the spatial phase shift distribution in components fabricated from pure lithographic resin (**a**) and lithographic resins containing 0.001% (**b**), 0.01% (**c**), and 0.1% (**d**) SiO_2_ nanoparticles. 3D reconstructions of material regions measuring 8.9 × 8.9 μm (**top right**), primary data (**bottom left**). The color scale represents the phase difference in transmitted radiation (red—maximum value, blue—minimum).

The internal structure of components fabricated from lithographic resin with and without SiO_2_ nanoparticles was investigated using modulation interference microscopy (Figure 4). The study examined regions of the components bounded by parallel polished planes. This technique can detect internal defects within components. If the refractive index of the medium differs from that of the nanoparticles, it becomes possible to obtain information about the spatial distribution of the nanoparticles inside the component. It was found that in samples without nanoparticles, regions with uniform phase shift were absent; the phase shift in each examined pixel was largely random. In components made from resin containing 0.001% SiO_2_ nanoparticles, regions with numerically similar phase shifts began to appear, measuring up to hundreds of nanometers in cross-section. In components made from resin containing 0.01% SiO_2_ nanoparticles, regions with numerically similar phase shifts measuring up to micrometers in cross-section were observed. In components made from resin containing 0.1% SiO_2_ nanoparticles, extended regions (up to 100 µm in length) with numerically similar phase shifts became apparent.

**Figure 5:**
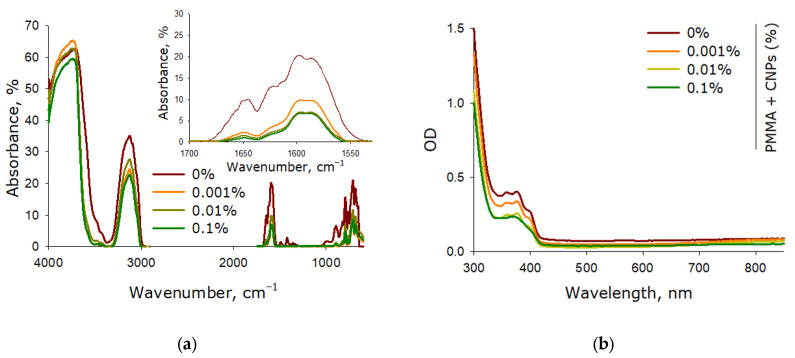
Optical properties of components fabricated from pure lithographic resin and lithographic resins containing 0.001%, 0.01%, and 0.1% SiO_2_ nanoparticles. FTIR absorption spectra of the samples (**a**); inset shows an enlarged spectral region with bands associated with carbon-carbon double bonds (C=C). Absorption spectra of the samples in the ultraviolet and visible ranges (**b**).

The optical properties of components made from pure lithographic resin and resins containing 0.001%, 0.01%, and 0.1% SiO_2_ nanoparticles were investigated. The study examined regions of the samples bounded by parallel polished planes. Fourier Transform Infrared Spectroscopy (FTIR) enables the study of composition and internal chemical structure (Figure 5a). The spectra of components fabricated from all studied resins showed bands characteristic of organic compounds. Components created through photopolymerization consistently exhibit carbon-carbon double bond (C=C) bands (1600–1650 cm^−1^). The intensity of these bands characterizes the degree of resin polymerization. Incorporating SiO_2_ nanoparticles into the resin reduced the amount of unreacted resin by nearly threefold.

The optical properties of the components in the ultraviolet and visible ranges were studied using differential double-beam UV-Vis spectrophotometer (Figure 5b). The investigation focused on sample regions bounded by parallel polished planes. It was found that both samples with and without SiO_2_ nanoparticles exhibited one absorption maximum (375 nm) and one absorption minimum (335 nm). The presence of nanoparticles, even at the highest concentration tested (0.1%), did not induce any changes in the absorption spectrum.

**Figure 6:**
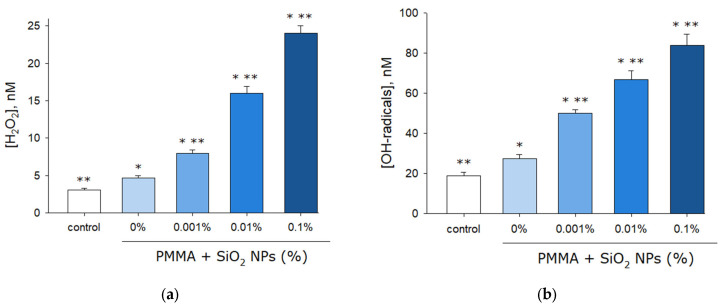
Effects of components fabricated from pure lithographic resin and lithographic resins containing 0.001%, 0.01%, and 0.1% SiO_2_ nanoparticles on the generation of hydrogen peroxide (H_2_O_2_) (**a**) and hydroxyl radicals (^•^OH) (**b**). Data are presented as mean values ± SEM (*n* = 3). *—significantly different from the control group (*p* < 0.05); **—significantly different from the group of samples without SiO_2_ nanoparticles (0%) (*p* < 0.05).

The effect of the plates made from lithographic resin with and without SiO_2_ nanoparticles on hydrogen peroxide formation in deionized water was investigated using the enhanced chemiluminescence method (Figure 6a). It was shown that in pure water (control), hydrogen peroxide forms at concentration of approximately 3 nM. When films made from resin without SiO_2_ nanoparticles (0%) are added to water, hydrogen peroxide is generated at concentration of about 5 nM. The addition of films made from resin containing 0.001% SiO_2_ nanoparticles results in hydrogen peroxide concentration slightly below 10 nM. With films containing 0.01% nanoparticles, the concentration increases to slightly above 15 nM, while films with 0.1% nanoparticles yield concentration slightly exceeding 25 nM.

We conducted additional experiments. Plate-shaped components (1 × 1 × 0.05 cm) of PMMA + 0.1% SiO_2_ NPs were placed in 20 mL of water. The sample was illuminated for 60 min with a 100-W incandescent lamp from a distance of 35 cm. The measured irradiance on the sample was 83.3 W/m^2^. In water without the sample, approximately 8 nM of hydrogen peroxide was formed. In water with the sample, just over 0.8 μM of hydrogen peroxide was formed. That is, in the presence of the PMMA + 0.1% SiO_2_ NPs sample in light, hydrogen peroxide formation in water is two orders of magnitude more intense.

The effect of the plates made from lithographic resin with and without SiO_2_ nanoparticles on hydroxyl radical formation in deionized water was investigated using the hydroxyl radical-specific fluorescent probe coumarin-3-carboxylic acid (CCA) (Figure 6b). It was shown that in pure water (control), hydroxyl radicals form at concentration of approximately 20 nM. When films made from resin without SiO_2_ nanoparticles (0%) are added to water, hydroxyl radicals are generated at concentration of about 30 nM. The addition of films containing 0.001% SiO_2_ nanoparticles produces hydroxyl radicals at concentration of approximately 50 nM. With films containing 0.01% nanoparticles, the concentration reaches slightly below 70 nM, while films with 0.1% nanoparticles yield concentration slightly under 90 nM.

**Figure 7:**
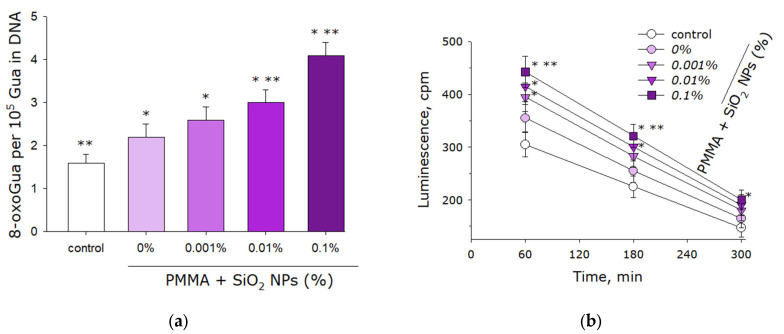
Effects of components fabricated from pure lithographic resin and lithographic resins containing 0.001%, 0.01%, and 0.1% SiO_2_ nanoparticles on the generation of 8-oxoguanine in DNA (**a**) and long-lived reactive protein species (**b**). Data are presented as mean values ± SEM (*n* = 3). *—significantly different from the control group (*p* < 0.05); **—significantly different from the group of samples without nanoparticles (0%) (*p* < 0.05).

The effect of the plates made from lithographic resin with and without SiO_2_ nanoparticles on the formation of 8-oxoguanine in DNA in vitro was investigated using an enzyme-linked immunosorbent assay (ELISA) with antibodies specific to 8-oxoguanine (Figure 7a). It was shown that in the absence of any polymers (control), approximately 1.5 molecules of 8-oxoguanine per 10^5^ guanine bases are formed in DNA. When films made from resin without SiO_2_ nanoparticles (0%) are added to DNA, approximately 2 molecules of 8-oxoguanine per 10^5^ guanine bases are generated. The addition of films containing 0.001% SiO_2_ nanoparticles results in approximately 2.5 molecules of 8-oxoguanine per 10^5^ guanine bases. With films containing 0.01% nanoparticles, the level increases to approximately 3 molecules of 8-oxoguanine per 10^5^ guanine bases, while with films containing 0.1% nanoparticles the level increases to 4 molecules.

The induction of long-lived reactive protein species by plates incorporating or lacking SiO_2_ nanoparticles was investigated via induced luminescence (Figure 7b). Control experiments without polymers demonstrated detectable formation of these protein species, exhibiting an initial luminescence intensity near 300 cpm. Introduction of resin plates without nanoparticles (0%) increased the initial luminescence to approximately 350 cpm. Progressive enhancement was observed with nanoparticle incorporation: 0.001% nanoparticles yielded intensities approaching 400 cpm, 0.01% resulted in values slightly above 400 cpm, and 0.1% produced intensities around 450 cpm. Across all experimental conditions, the measured half-life of the protein species consistently approximated 4 h.

**Figure 8:**
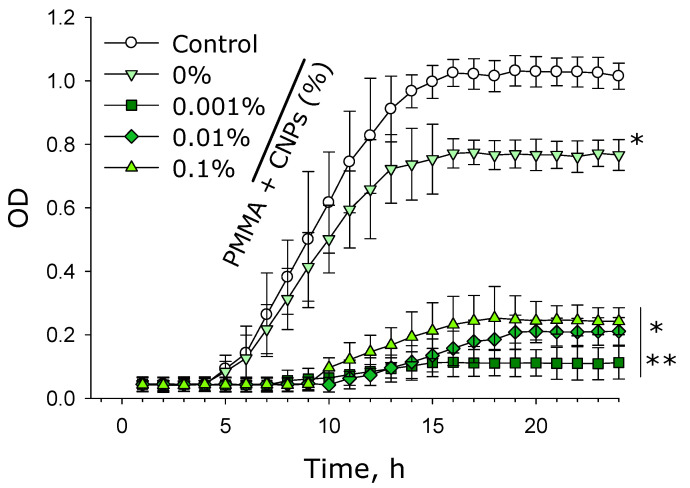
Effects of components fabricated from pure lithographic resin and lithographic resins containing 0.001%, 0.01%, and 0.1% SiO_2_ nanoparticles on the growth and development of E. coli suspension cultures. Data are presented as mean values ± standard error of the mean (*n* = 3). *—significantly different from the control group (*p* < 0.05); **—significantly different from the group of samples without nanoparticles (0%) (*p* < 0.05).

The effect of the plates made from photolithographic resins with and without SiO_2_ nanoparticles on the growth and development of *E. coli* bacteria in suspension cultures was studied (Figure 8). It was shown that in the absence of any photolithographic resin components (control), the lag phase duration was approximately 4 h, followed by a logarithmic phase from 4 to 16 h. Upon reaching the stationary phase, the optical density of the bacterial culture was about 1.0 unit. In the presence of the plates made from resin without nanoparticles, the durations of the lag and logarithmic phases remained unchanged and were identical to those in the control group. However, during the stationary phase, the optical density recorded in the culture medium was approximately 0.75 units. For components with 0.001% SiO_2_ nanoparticles, the lag phase lasted 8 h and the logarithmic phase—9 h. With components containing 0.01% and 0.1% SiO_2_ nanoparticles, the lag phase also persisted for 8 h, while the logarithmic phase duration was 8 h. In these cases, the optical density during the stationary phase was approximately 0.2 units.

**Figure 9:**
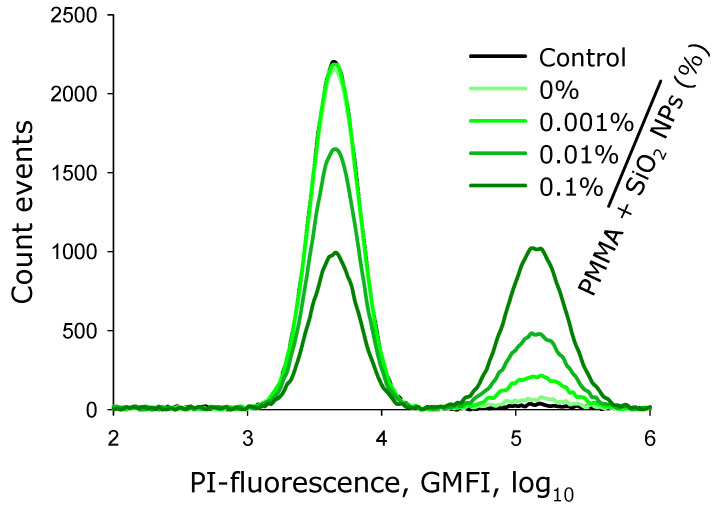
Histograms showing the distribution of bacterial cells *E. coli* by geometric mean fluorescence intensity of propidium iodide after incubation with the plates fabricated from pure lithographic resin and lithographic resins containing 0.001%, 0.01%, and 0.1% SiO_2_ nanoparticles.

Flow cytometry was used to investigate the effects of the plates fabricated from pure lithographic resin and resins containing 0.001%, 0.01%, and 0.1% SiO_2_ nanoparticles on the viability of *E. coli* bacteria (Figure 9). The left gate represents the population of viable cells analyzed, while the right gate identifies the propidium iodide (PI)-positive (non-viable) cells. The analysis settings allowed for clear discrimination between viable and non-viable cells. It was established that under control conditions (without lithographic resin components) and in the presence of components without nanoparticles, no significant increase in the proportion of dead bacterial cells was observed. In all other experimental groups, an increase in the percentage of PI-positive cells was detected.

**Figure 10:**
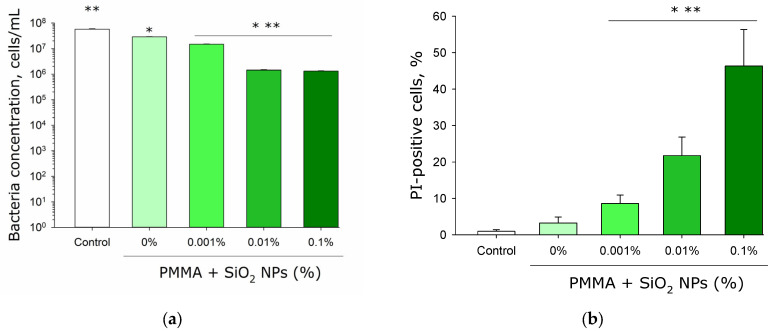
Effect of the plates fabricated from pure lithographic resin and lithographic resins containing 0.001%, 0.01%, and 0.1% SiO_2_ nanoparticles on the concentration of *E. coli* bacteria in the culture medium (**a**); proportion of non-viable cells in the culture medium (**b**). Cultivation time: 24 h. Data were obtained by flow cytofluorometry. Data are presented as mean values ± standard deviation (*n* = 3). *—significantly different from the control group (*p* < 0.05); **—significantly different from the group of samples without nanoparticles (0%) (*p* < 0.05).

Analysis of histograms obtained by flow cytofluorometry enabled both numerical determination of *E. coli* concentration in the culture medium and quantification of the non-viable bacterial fraction. Under control conditions, approximately 6 × 10^7^ cells/mL of *E. coli* were detected in the culture medium after 24 h of incubation (Figure 10a). In the presence of flat components made from photopolymer resin, about 3 × 10^7^ cells/mL were detected. Culture medium containing components with 0.001% SiO_2_ nanoparticles showed approximately 1 × 10^7^ cells/mL. With components containing 0.01% and 0.1% SiO_2_ nanoparticles, the culture medium contained fewer than 2 × 10^6^ cells/mL.

The lowest proportion of non-viable bacteria was observed in the control culture medium and in medium containing components without SiO_2_ nanoparticles (Figure 10b). In the presence of the plates made from photolithographic resin with 0.001% SiO_2_ nanoparticles, the proportion of non-viable bacterial cells exceeded 8%. The presence of the plates with 0.01% nanoparticle concentration caused death of more than 20% of the total cell population. Plates with 0.1% nanoparticle concentration resulted in death of over 45% of all cells in the medium.

**Figure 11:**
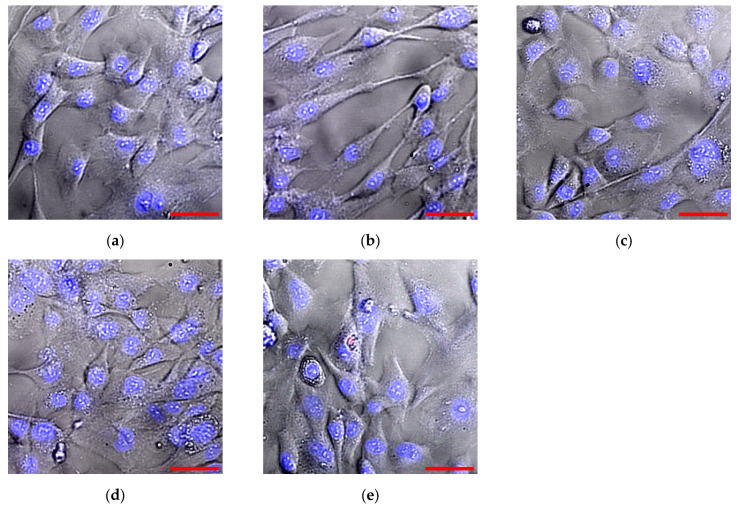
HSF cell morphology following 72-h in vitro cultivation: control group (**a**), with lithographic resin plate without nanoparticles (**b**); with plates containing 0.001 (**c**), 0.01 (**d**), and 0.1% (**e**) SiO_2_ nanoparticles. Scale bar: 10 μm (lower right).

The possibility of cultivating HSF mammalian cells on the surface of flat components fabricated from photolithographic resins with and without silicon dioxide nanoparticles was investigated. Microscopic examination of cell morphology revealed no significant differences among all experimental groups (Figure 11). In all experimental groups, cells adhered and spread on the surfaces with comparable efficiency. No differences were detected between cultivation on culture glass and plates fabricated from resins with or without SiO_2_ nanoparticles.

**Figure 12:**
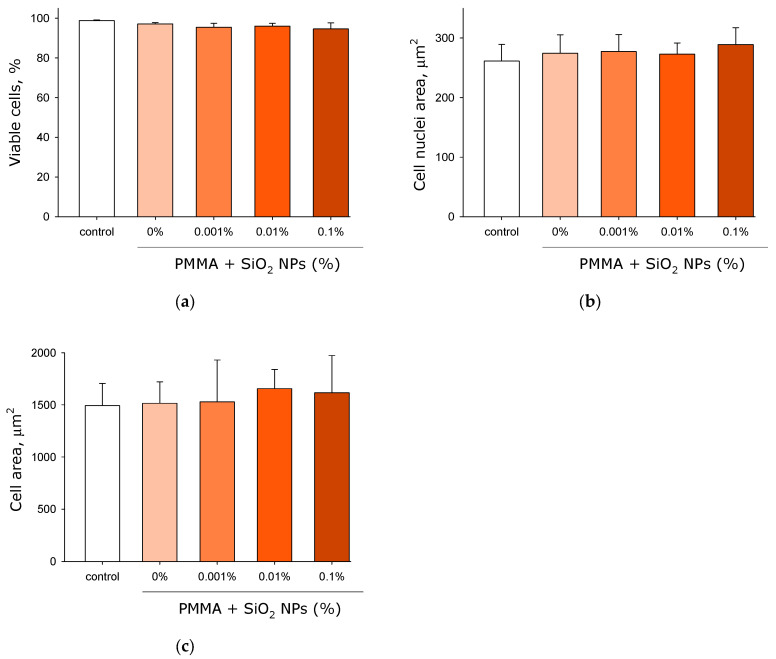
Effect of the plates fabricated from pure lithographic resin and lithographic resins containing 0.001%, 0.01%, and 0.1% SiO_2_ nanoparticles on eukaryotic HSF cell growth and development: proportion of viable cells in cultures (**a**); nuclear area (**b**); cell area (**c**). Data are presented as mean values ± standard deviation (*n* = 3).

Fluorescence microscopy analysis revealed high cell viability (>95%) across all test conditions, with minimal non-viable cell counts observed in every experimental group (Figure 12a). All experimental groups showed statistically similar viability rates. Alterations in nuclear size often serve as an indicator of long-term cellular stress. Fluorescence microscopy was used to investigate the effects of the plates fabricated from pure lithographic resin and resins containing 0.001%, 0.01%, and 0.1% SiO_2_ nanoparticles on HSF cell nuclear area (Figure 12b). Nuclear areas showed no significant differences across all experimental groups. Image analysis confirmed average HSF nuclear size of 250–300 μm^2^. Similarly, the effects on total HSF cell area were examined (Figure 12c). Cell areas remained consistent across all test conditions, with average cell area of 1500 μm^2^ observed in all groups.

## 3. Discussion

Currently, SiO_2_ nanoparticles are primarily synthesized through conventional chemical methods [37] and plasma chemistry [38], including laser-induced processes [39]. Laser-based nanoparticle synthesis is an environmentally friendly approach (no chemical reagents required) and offers precise control [40]. In this work, nanoparticles were produced by laser ablation of silicon dioxide plate in deionized water. It should be noted that the nanoparticle preparation exhibits a relatively narrow size distribution and uniform ζ-potential (Figure 1a,b,d). The obtained absorption spectrum of the aqueous nanoparticle colloid (Figure 1c) fully corresponds to the characteristic absorption profile of silicon dioxide nanoparticles in water [41].

A method for incorporating silicon dioxide nanoparticles into photolithographic resin was developed. The composite resin containing 0.1% nanoparticles enabled the fabrication of complex scaffold samples via additive manufacturing with maximum achievable resolution of 0.05 mm (Figure 2). In addition to scaffolds, we produced thin, round, optically transparent plates that were used in most tests.

Defects—both on the surface and internal—often arise during the fabrication of components from nanocomposite resins using additive manufacturing or photopolymerization [42]. Nanofillers in resins for additive manufacturing frequently complicate post-processing [43] and impede high-quality surface polishing [44]. In contrast, the components fabricated in this study from PMMA-based composite resin with SiO_2_ nanoparticles demonstrated excellent suitability for polishing. Polishing of components containing 0.1% nanoparticles achieved height variation of less than 10 nm over 10 × 10 μm^2^ area (Figure 3).

When using composite lithographic resins in additive manufacturing or photopolymerization, nanoparticle aggregation is frequently observed [45]. Unsurprisingly, this uneven nanoparticle distribution is also evident in the final products [46,47]. The components fabricated in this study were free from defects, including optical imperfections. However, modulation interference microscopy revealed regions with similar optical phase shift values within the manufactured components (Figure 4). The microscope’s laser operates at a wavelength of 405 nm. The refractive index of the PMMA-based photolithographic resin is 1.50 at this wavelength [48], while that of silicon dioxide is 1.46 [49]. The refractive index difference of approximately 0.04 is sufficient to detect areas with higher and lower nanoparticle concentrations within the components. Interestingly, extended regions approaching macroscopic dimensions were identified in the fabricated structures.

Under the influence of ultraviolet radiation, the polymerization process begins in the lithographic resin, due to which it hardens [50]. Since the photo-curing process is largely stochastic, there are always components in the resin that have not polymerized [51]. An increase in the concentration of components not involved in the polymerization process affects, on the one hand, the physico-chemical properties of the final product [52], on the other hand, negatively affects the toxicological characteristics of the product [53]. It has been shown that SiO_2_ nanoparticles increase the degree of polymerization of PMMA-like resin under the action of ultraviolet radiation (Figure 5). It is known that SiO_2_ nanoparticles generate reactive oxygen species (ROS) under the influence of light [54]. Hydrogen peroxide, one of the ROS, is used for post-treatment of components manufactured using MSLA technology in order to further polymerize possible resin residues on the surface [55]. It is possible that these properties of SiO_2_ nanoparticles have the main effect on the degree of polymerization of resins during the additive process or photopolymerization.

It has been shown that when samples fabricated from lithographic resin containing SiO_2_ nanoparticles are added to water, significant additional generation of hydrogen peroxide and hydroxyl radicals is observed (Figure 6). It is known that SiO_2_ nanoparticles participate in the photogeneration of ROS [56]. It is important to note that the process of ROS generation described in this work occurs in the dark, not under the action of light. Only one study was found that may indirectly indicate that silicon nanoparticles lead to the generation of ROS in aqueous solutions without additional illumination [57]. At the same time, it is widely known that SiO_2_ nanoparticles cause the generation of ROS in cells and tissues [58,59,60]. SiO_2_ nanoparticles enhance the formation of ROS under the action of ionizing radiation and are, in fact, radiosensitizers [61].

The development of oxidative stress is usually associated with an increase in ROS concentration beyond the capacity of antioxidant systems [62]. Oxidative stress causes oxidative damage to various biomolecules, including DNA [63], protein molecules [64], and lipid molecules [65]. High-intensity oxidative stress is the main cause of cell death from exposure to lethal doses of ionizing radiation [66]. Typically, the level of oxidative stress, and consequently the level of damage to biomacromolecules, can be modulated by various low-molecular-weight and high-molecular-weight antioxidants [67]. This work demonstrated that when plates made of photolithographic resin containing SiO_2_ nanoparticles come into contact, more intensive formation of 8-oxoguanine in DNA occurs compared to the control (Figure 7a)—a key marker of oxidative stress. Also, plates made of photolithographic resin with SiO_2_ nanoparticles lead to more intensive formation of long-lived reactive protein species compared to the control (Figure 7b). It is known that under the action of heat, amino acids are able to reduce DNA damage, including the rate of 8-oxoguanine formation in it [68]. It was previously shown that hyperthermia leads to the formation of long-lived reactive protein species in aqueous protein solutions. Such long-lived reactive protein species are capable of long-term generation of ROS after hyperthermia [69]. Furthermore, long-lived reactive protein species are effectively eliminated by some low-molecular-weight antioxidants [70], which leads to a reduction in the overall level of oxidative stress [71]. It should be noted that relatively high concentrations of ROS do not always cause negative consequences in living systems; sometimes they can have a stimulating effect on them [72].

It has been shown that lithographic PMMA-like resins with SiO_2_ nanoparticles inhibit both the growth and development of *E. coli* cells (Figure 8). Antibacterial effect is also observed in the presence of the plates made from lithographic resins with SiO_2_ nanoparticles, as a portion of the bacteria become non-viable (Figure 9 and Figure 10). The antibacterial effect of SiO_2_ nanoparticles was established over a decade ago [73]. According to the literature, the antibacterial properties of SiO_2_ nanoparticles are realized through contact-based destruction [74], accumulation and alteration of bacterial cell wall properties [75], inactivation of membrane transport [76], and the induction of endogenous oxidative stress [77]. Despite the significant antibacterial effect, incubation with the plates made from resins containing SiO_2_ nanoparticles did not lead to changes in the morphology and growth parameters of mammalian cells (Figure 11 and Figure 12). The development of mammalian cells on plates with nanoparticles proceeded normally. It is possible that the resins with SiO_2_ nanoparticles obtained in this study could be a material for products used in biomedicine, pharmacology, and the food industry.

## 4. Materials and Methods

### 4.1. Synthesis of SiO_2_ Nanoparticles

Silicon dioxide nanoparticles were obtained by laser ablation, and high-purity silicon oxide (99.99% purity) was used as a massive target [78]. The target was clamped to the bottom of the flow reactor. The reactor was filled with deionized water so that the target was completely covered with water. The thickness of the deionized water layer between the atmosphere and the target was about 2–3 mm. The total amount of water in the flow reactors, tubes, compensating tank and pump is 100 mL. A P-Mark TT 100 fiber ytterbium laser (Pokkels, Moscow, Russia) was used to generate SiO_2_ nanoparticles. Characteristics of the laser source: 1064 nm, 35 kHz, 200 ns and 1.5 mJ. The laser beam was focused on silicon dioxide target. The beam was moved over the target surface using the LScanH galvano-optical scanning system (Ateko-TM, Moscow, Russia) with an F-Theta lens (focal length—90 mm). The speed of the beam was 3 m/s, the mixing trajectory was set of straight lines inscribed through 20 microns into a rectangular area with 15 × 25 mm.

### 4.2. Methods of Characterization of SiO_2_ Nanoparticles

The hydrodynamic diameter, ζ-potential, and concentration of SiO_2_ nanoparticles in aqueous colloid were characterized using dynamic light scattering on a Malvern Zetasizer Ultra analyzer (Malvern Panalytical Ltd., Malvern, UK). UV-Vis spectra of the SiO_2_ nanoparticle aqueous colloids were acquired using a Cintra 4040 differential double-beam spectrophotometer (GBC Scientific Equipment Pty Ltd., Keysborough, VIC, Australia). The morphology of the SiO_2_ nanoparticles was examined using a Libra 200 FE HR transmission electron microscope (TEM) (Carl Zeiss, Jena, Germany).

### 4.3. The Method of Introducing SiO_2_ Nanoparticles into Photolithographic Resin

The water in the colloid of SiO_2_ nanoparticles was changed to acetone. For this purpose, an aqueous colloid of SiO_2_ nanoparticles was centrifuged for 20 h at approximately 15.300 rpm (21.984× *g*) using 3–16 KL centrifuge (Sigma-Aldrich, St. Louis, MO, USA). The filler liquid was drained, and instead of the filler liquid consisting of water, acetone in the same amount was added to the SiO_2_ nanoparticles. To transfer SiO_2_ nanoparticles from the precipitate to the colloid, they were exposed to ultrasound (40 kHz, 3 min) and shaken intensively on a shaker (2000 rpm, 5 min). The procedure was performed several times in a row, until the dehydrated colloid was obtained. Colloid of SiO_2_ nanoparticles in acetone was stored in an airtight glass vial in the dark. Before use, the prepared colloid was mixed with photolithographic PMMA-like resin Dental Clear PRO (Harz Labs, Mytischi, Russia). After mixing, the resin was shaken intensively on a shaker (2000 rpm, 5 min), and then exposed to ultrasound (40 kHz, 3 min). The concentration of nanoparticles in the resin was 0.001%, 0.01%, or 0.1% by weight.

### 4.4. Additive Manufacturing of Composite Photolithographic Resin Samples

Fabrication was carried out using a Saturn 3 Ultra 12K MSLA printer (Elegoo, Shenzhen, China) with photolithographic resins containing 0, 0.001%, 0.01%, or 0.1% SiO_2_ nanoparticles. All printed samples subsequently underwent post-processing. After fabrication, the samples were intensively rinsed in isopropanol for 6 min, followed by immersion in an ultrasonic isopropanol bath for another 6 min. Subsequently, the parts were dried, and the surfaces of the dry parts were treated with a triatomic alcohol (glycerol) and placed in a UW-02 curing unit (Creality3D, Shenzhen, China) for 30 min. After this step, the samples were again immersed in an ultrasonic isopropanol bath for 6 min, followed by drying in a thermal chamber for 30 min at 80 °C. Prior to investigations, the parts were stored in sealed containers at room temperature.

### 4.5. Methods of Characterization of Composite Photolithographic Resin Samples

The micro- and nanostructure of the surface of the manufactured samples was characterized using an atomic force microscope NT-MDT microscope (LLC, Zelenograd, Russia). The optical properties of parts made of composite photolithographic resins were characterized using the IR-8000 FTIR spectrometer (SAS LLC, Krasnoyarsk, Russia) with the ZnSe Sealed Flat Plate prefix (Pike Technologies, WI, USA), as well as the Cintra 4040 dual-beam differential UV/Vis spectrometer (GBC Scientific Equipment Pty Ltd., Victoria, Australia). The distribution of nanoparticles in the volume of parts made of composite photolithographic resins was characterized using a MIM-321 modulation interference microscope (Amphora laboratories, Moscow, Russia).

### 4.6. Methods for Measuring the Concentration of Reactive Oxygen Species

The influence of plate-shaped components (1 × 1 × 0.05 cm) on the generation of reactive oxygen species, specifically hydrogen peroxide and hydroxyl radicals, was investigated. The concentration of hydrogen peroxide in the aqueous environment was characterized using the enhanced chemiluminescence method. Chemiluminescence detection was performed with highly sensitive “Biotoks-7A-USE” chemiluminometer (Engineering Center-Ecology, Moscow, Russia). The chemiluminescence reaction solution consisted of Tris-HCl buffer supplemented with luminol, 4-iodophenol, and horseradish peroxidase enzyme. The plates in antistatic vials (20 mL) were incubated for 3 h at 40 °C in liquid ultra-thermostat. The method sensitivity was 0.1 nM hydrogen peroxide. All experimental procedures are described in detail previously in [68].

Hydroxyl radicals were detected using the fluorescent probe coumarin-3-carboxylic acid. Components in vials (20 mL) were incubated for 2 h at 80 °C. Subsequently, the solution in the vials was alkalized using Tris-HCl buffer to pH 8.5. The resulting hydroxylation product of coumarin-3-carboxylic acid—7-hydroxycoumarin-3-carboxylic acid—was detected using a JASCO 8300 spectrofluorometer (JASCO, Tokyo, Japan) (λ_ex_ = 400 nm, λ_em_ = 450 nm). All experimental procedures are described in detail previously in [38].

### 4.7. Enzyme Immunoassay for the Determination of the Amount of 8-Oxoguanine in DNA

The amount of 8-oxoguanine molecules in DNA was characterized using the method of enzyme immunoassay with monoclonal antibodies. The experiments used samples in the form of thin plates measuring 1 × 1 × 0.05 cm. The plates were added to a DNA solution (10 mL) and incubated for 180 min at 40 °C. The dilution of primary antibodies to 8-oxoguanine is about 1:2000, the dilution of secondary antibodies is about 1:1000. The measurements were carried out on a Feyond-A400 plate photometer (Allsheng, Hangzhou, China) at wavelength of 405 nm. All experimental procedures were described in detail earlier in [68].

### 4.8. Method for Determining the Number of Long-Lived Reactive Protein Forms (LRPS)

In the experiments, samples in the form of plates (1 × 1 × 0.05 cm) were used. The samples were introduced into an aqueous BSA colloid (10 mL, 0.1%) immediately before the start of the experiment and incubated for 120 min at 40 °C. Before measuring, the colloid was left for 30 min in a dark room at room temperature. LRPS were detected by induced chemiluminescence of protein colloids. Luminescence was detected with a highly sensitive Biotox-7A chemiluminometer (Engineering Center—Ecology, Moscow, Russia). All experimental procedures were described in detail earlier in [79].

### 4.9. Method for Studying E. coli Growth Curves

In the experiments, samples in the form of plates (1 × 1 × 0.05 cm) were used. The parts were sterilized before the experiment. The experiments were carried out in 24-well plates. 1 mL of LB culture medium with *E. coli* BL21 (DE3) (Eurogen, Moscow, Russia) was added to each well of the tablet. A tablet without a lid was placed in tablet photometer incubator. The culture was measured and mixed automatically 1 time per hour at a constant temperature of 37 °C. All experimental procedures were described in detail earlier in [80].

### 4.10. Flow Cytofluorometry Method

Flow cytometry was performed on a Longcyte cytofluorimeter (ChBio, Nantou, Taiwan). *E. coli* preparation and cultivation were performed according to the procedure described above. Bacterial staining was performed using 1 mL of propidium iodide (PI) dissolved in a phosphate-salt buffer at a concentration of 4 µM. For complete staining, the bacterial culture was incubated with propidium iodide in the dark for at least 60 min. To assess the intensity of the fluorescence of PI, both lateral and forward light scattering (λ_ex_ = 535 nm, λ_em_ = 617 nm) were measured. The number of bacteria in the suspension was determined by automated recalculation of the number of events per 1 mL of suspension. The proportion of dead cells was estimated by the threshold method. All experimental procedures were described in detail earlier [81].

### 4.11. Method for Assessing Toxicity to Eukaryotic Cell Culture

The cytocompatibility of surfaces from the samples fabricated with photolithographic resins, both with and without SiO_2_ nanoparticles, was studied using a culture of human spleen fibroblasts (HSF) (ATCC line No. PCS-201-012). Cells were cultured under standard conditions using DMEM/F12 nutrient medium. The medium was supplemented with 10% fetal bovine serum, 2 mM L-glutamine, 25 U/mL penicillin, and 25 µg/mL streptomycin. Cultivation was carried out in a CO_2_ incubator for 72 h. Cell viability was assessed by fluorescence microscopy. Cell staining was performed using the fluorescent dyes Hoechst 33342 (for nuclear staining), Rhodamine (for visualization of mitochondria), and propidium iodide (for dead cells detection). Cell specimens were examined using a Leica DMI 4000B microscopic system (Leica, Wetzlar, Germany), and microphotographs were processed with ImageJ software v. 1.54p. All experimental procedures are described in detail previously [82].

## 5. Conclusions

As a result of this study, new composite photolithographic resin based on polymethyl methacrylate modified with SiO_2_ nanoparticles synthesized by laser ablation was successfully developed and characterized. The key achievement is the demonstration of the full technological compatibility of this nanocomposite with the additive manufacturing process, enabling the fabrication of complex structures, including scaffolds and optically transparent plates, with high resolution and excellent polishability. Importantly, despite the identification of areas with non-uniform distribution of nanoparticles, the finished products were free of macroscopic defects. The key result of this study was the establishment of the dual functional role of SiO_2_ nanoparticles in the polymer matrix. First, they were shown to act as an effective polymerization enhancer, increasing the degree of resin curing under UV radiation, potentially improving the physicochemical and toxicological properties of the final product. Secondly, and most significantly, the nanoparticles impart to the material pronounced and continuous activity in generating reactive oxygen species (ROS), including hydrogen peroxide and hydroxyl radicals upon contact with an aqueous environment. Oxidative damage to key biomolecules, such as DNA and proteins, was observed upon incubation with the resulting materials. Significant antibacterial effect against E. coli cells was also demonstrated. Furthermore, the material’s cytocompatibility with mammalian cells was high, as evidenced by the absence of negative impact on their morphology and proliferation. Thus, the resulting material implements the principle of selective toxicity, being lethal to bacterial cells but safe for animal cells. Taken together, the results confirm the potential of the developed composite material for the 3D printing of complex functional components intended for use in applications critical to sterility and biocompatibility. These include biomedicine (implants, surgical instruments, diagnostic system components), pharmaceuticals (drug delivery systems), and the food industry (packaging, process equipment components). The combination of additive manufacturing technology with the material’s inherent antimicrobial properties and safety opens up new possibilities for the customized production of products with specified functional characteristics.

## Data Availability

The raw data supporting the conclusions of this article will be made available by the authors on request.

## References

[1] Khan W.S., Asmatulu E., Asmatulu R. (2025). Nanotechnology Emerging Trends, Markets and Concerns. Nanotechnology Safety.

[2] Ramakoti I.S., Panda A.K., Gouda N. (2023). A Brief Review on Polymer Nanocomposites: Current Trends and Prospects. J. Polym. Eng..

[3] Molkova E.A., Pustovoi V.I., Baimler I.V., Simakin A.V., Burmistrov D.E., Gorudko I.V., Gudkov S.V. (2024). Optical Study of the Influence of the Medium Acidity on the Interaction between Gold Nanoparticles and Bovine Serum Albumin in Aqueous Solution. Phys. Wave Phen..

[4] Gudkov S.V., Sarimov R.M., Astashev M.E., Pishchalnikov R.Y., Yanykin D.V., Simakin A.V., Shkirin A.V., Serov D.A., Konchekov E.M., Gusein-zade N.G. (2024). Modern Physical Methods and Technologies in Agriculture. Phys. Usp..

[5] Nastulyavichus A., Khaertdinova L., Tolordava E., Yushina Y., Ionin A., Semenova A., Kudryashov S. (2022). Additive Nanosecond Laser-Induced Forward Transfer of High Antibacterial Metal Nanoparticle Dose onto Foodborne Bacterial Biofilms. Micromachines.

[6] Satchanska G., Davidova S., Petrov P.D. (2024). Natural and Synthetic Polymers for Biomedical and Environmental Applications. Polymers.

[7] Nastulyavichus A., Kudryashov S., Tolordava E., Rudenko A., Kirilenko D., Gonchukov S., Ionin A., Yushina Y. (2022). Generation of silver nanoparticles from thin films and their antibacterial properties. Laser Phys. Lett..

[8] Sukhareva K., Chernetsov V., Burmistrov I. (2024). A Review of Antimicrobial Polymer Coatings on Steel for the Food Processing Industry. Polymers.

[9] Krishna R.H., Chandraprabha M.N., Monika P., Br T., Chaudhary V., Manjunatha C. (2022). Biomolecule Conjugated Inorganic Nanoparticles for Biomedical Applications: A Review. Biotechnol. Genet. Eng. Rev..

[10] Oruganti S.K., Karras D., Thakur S.S., Nagpal K., Gupta S.K. (2024). Case Studies on Holistic Medical Interventions.

[11] Balderas-León I., Silva-Jara J.M., López-Álvarez M.Á., Ortega-Gudiño P., Barrera-Rodríguez A., Neri-Cortés C. (2024). Degradation of Malachite Green Dye by Solar Irradiation Assisted by TiO_2_ Biogenic Nanoparticles Using Vaccinium Corymbosum Extract. Sustainability.

[12] Burmistrov D.E., Simakin A.V., Smirnova V.V., Uvarov O.V., Ivashkin P.I., Kucherov R.N., Ivanov V.E., Bruskov V.I., Sevostyanov M.A., Baikin A.S. (2021). Bacteriostatic and Cytotoxic Properties of Composite Material Based on ZnO Nanoparticles in PLGA Obtained by Low Temperature Method. Polymers.

[13] Hashemi S.-S., Alizadeh R., Rafati A., Mohammadi A., Mortazavi M., Hashempur M.H. (2025). Investigation of Silicon Oxide Nanoparticle-Enhanced Self-Healing Hydrogel for Cartilage Repair and Regeneration in Rabbit Earlobe Models. J. Drug Target..

[14] Xu Z., Li L., Liu Y., Yang R. (2025). Photodegradation Behavior of Nanosilica-Filled PMMA Composite: Cooperative Effect of Mixed Solvents and Interfacial Functional Groups. Polymers.

[15] Toledo-Manuel I., Pérez-Alvarez M., Cadenas-Pliego G., Cabello-Alvarado C.J., Tellez-Barrios G., Ávila-Orta C.A., Ledezma-Pérez A.S., Andrade-Guel M., Bartolo-Pérez P. (2025). Sonochemical Functionalization of SiO_2_ Nanoparticles with Citric Acid and Monoethanolamine and Its Remarkable Effect on Antibacterial Activity. Materials.

[16] Lamb D.R. (1970). Some Electrical Properties of the Silicon-Silicon Dioxide System. Thin Solid Films.

[17] Hahn P.O. (2012). Sand and Silicon. Science That Changed the World. By Denis McWhan. Angew. Chem. Int. Ed..

[18] Gmoshinski I.V., Shipelin V.A., Khotimchenko S.A. (2018). Nanomaterials in Food Products and Their Package: Comparative Analysis of Risks and Advantages. Health Risk Anal..

[19] Sampaio F.C., de Oliveira A.F.B., Fernandes N.L.S., Gentile A.C.C., Marinho G.B., Bönecker M.J.S., Paschoal M.A.B., D’Alpino P.H.P., Vilhena F.V. (2024). Silicon-, Silica-, and Silicate-Toothpastes for Remineralization and Repair of Teeth: A Scoping Review. Oral.

[20] Qian K.K., Bogner R.H. (2012). Application of Mesoporous Silicon Dioxide and Silicate in Oral Amorphous Drug Delivery Systems. J. Pharm. Sci..

[21] Pelin G., Sonmez M., Pelin C.-E. (2024). The Use of Additive Manufacturing Techniques in the Development of Polymeric Molds: A Review. Polymers.

[22] Ramos A., Angel V.G., Siqueiros M., Sahagun T., Gonzalez L., Ballesteros R. (2025). Reviewing Additive Manufacturing Techniques: Material Trends and Weight Optimization Possibilities Through Innovative Printing Patterns. Materials.

[23] Kumar S.A., Prasad R.V.S. (2021). Basic Principles of Additive Manufacturing: Different Additive Manufacturing Technologies. Additive Manufacturing.

[24] Zhou L., Miller J., Vezza J., Mayster M., Raffay M., Justice Q., Al Tamimi Z., Hansotte G., Sunkara L.D., Bernat J. (2024). Additive Manufacturing: A Comprehensive Review. Sensors.

[25] Rutkay B., Laliberté J. (2016). Design and Manufacture of Propellers for Small Unmanned Aerial Vehicles. J. Unmanned Veh. Sys..

[26] Erkoc P., Ulucan-Karnak F. (2021). Nanotechnology-Based Antimicrobial and Antiviral Surface Coating Strategies. Prosthesis.

[27] Culbreath C.J., Taylor M.S., McCullen S.D., Mefford O.T. (2024). A Review of Additive Manufacturing in Tissue Engineering and Regenerative Medicine. Biomed. Mater. Devices.

[28] Segneanu A.-E., Bejenaru L.E., Bejenaru C., Blendea A., Mogoşanu G.D., Biţă A., Boia E.R. (2025). Advancements in Hydrogels: A Comprehensive Review of Natural and Synthetic Innovations for Biomedical Applications. Polymers.

[29] Mekha K., Abu T.Y.N., Sudhakar K., Zainol N., Hasan N., Abdul Karim M.S. (2024). Development of Sustainable Polymer-Based Dielectric Composites from Agricultural Waste: A Review. Heliyon.

[30] Singha A.S., Thakur V.K. (2009). Chemical Resistance, Mechanical and Physical Properties ofBiofibers-Based Polymer Composites. Polym. Plast. Technol. Eng..

[31] Zafar M.S. (2020). Prosthodontic Applications of Polymethyl Methacrylate (PMMA): An Update. Polymers.

[32] Alobaidani A.D., Furniss D., Johnson M.S., Endruweit A., Seddon A.B. (2010). Optical Transmission of PMMA Optical Fibres Exposed to High Intensity UVA and Visible Blue Light. Opt. Lasers Eng..

[33] Gross S., Camozzo D., Di Noto V., Armelao L., Tondello E. (2007). PMMA: A Key Macromolecular Component for Dielectric Low-κ Hybrid Inorganic–Organic Polymer Films. Eur. Polym. J..

[34] Xinlong M., Yang Y., Jianxiong M., Xiaohong W., Yanjun Z. (2010). Comparison of Mechanical Properties of Polymethyl Methacrylate of Different Mixing Ratios. J. Med. Eng. Technol..

[35] Evchuk I.Y., Musii R.I., Makitra R.G., Pristanskii R.E. (2005). Solubility of Polymethyl Methacrylate in Organic Solvents. Russ. J. Appl. Chem..

[36] Chang C., Merritt K. (1992). Microbial Adherence on Poly(Methyl Methacrylate) (PMMA) Surfaces. J. Biomed. Mater. Res..

[37] Zarei V., Mirzaasadi M., Davarpanah A., Nasiri A., Valizadeh M., Hosseini M.J.S. (2021). Environmental Method for Synthesizing Amorphous Silica Oxide Nanoparticles from a Natural Material. Processes.

[38] Bulychev N.A. (2022). Synthesis of Gaseous Hydrogen and Nanoparticles of Silicon and Silicon Oxide by Pyrolysis of Tetraethoxysilane in an Electric Discharge under the Action of Ultrasound. Int. J. Hydrog. Energy.

[39] Kuzmin P.G., Shafeev G.A., Bukin V.V., Garnov S.V., Farcau C., Carles R., Warot-Fontrose B., Guieu V., Viau G. (2010). Silicon Nanoparticles Produced by Femtosecond Laser Ablation in Ethanol: Size Control, Structural Characterization, and Optical Properties. J. Phys. Chem. C.

[40] Coviello V., Reffatto C., Fawaz M.W., Mahler B., Sollier A., Lukic B., Rack A., Amans D., Amendola V. (2025). Time-Resolved Dynamics of Laser Ablation in Liquid with Gas-Evolving Additives: Toward Molding the Atomic Structure of Nonequilibrium Nanoalloys. Adv. Sci..

[41] Barachevsky V.A., Kobeleva O.I., Gorelik A.M., Krayushkin M.M. (2018). Spectral Manifestations of the Interaction of Silicon Dioxide Nanoparticles with Molecules of Photochromic Compounds. Opt. Spectrosc..

[42] Billings C., Cai C., Liu Y. (2021). Utilization of Antibacterial Nanoparticles in Photocurable Additive Manufacturing of Advanced Composites for Improved Public Health. Polymers.

[43] Krishna J.S., Chaudhary V., Mehta J., Malhotra P., Gupta S., Gupta P. (2022). Synergistic Reinforcement of Nanofillers in Biocomposites Developed by Additive Manufacturing Techniques. Biomass Conv. Bioref..

[44] Erturk-Avunduk A.T., Atılan-Yavuz S., Filiz H., Cengiz-Yanardag E. (2024). A Comparative Study of Polishing Systems on Optical Properties and Surface Roughness of Additively Manufactured and Conventional Resin Based Composites. Sci. Rep..

[45] Gil L.D., Monteiro S.N., Colorado H.A. (2024). Polymer Matrix Nanocomposites Fabricated with Copper Nanoparticles and Photopolymer Resin via Vat Photopolymerization Additive Manufacturing. Polymers.

[46] Khan Y., Sadia H., Ali Shah S.Z., Khan M.N., Shah A.A., Ullah N., Ullah M.F., Bibi H., Bafakeeh O.T., Khedher N.B. (2022). Classification, Synthetic, and Characterization Approaches to Nanoparticles, and Their Applications in Various Fields of Nanotechnology: A Review. Catalysts.

[47] Elmowafy M., Shalaby K., Elkomy M.H., Alsaidan O.A., Gomaa H.A.M., Abdelgawad M.A., Mostafa E.M. (2023). Polymeric Nanoparticles for Delivery of Natural Bioactive Agents: Recent Advances and Challenges. Polymers.

[48] Beadie G., Brindza M., Flynn R.A., Rosenberg A., Shirk J.S. (2015). Refractive Index Measurements of Poly(Methyl Methacrylate) (PMMA) from 04–16 μm. Appl. Opt..

[49] Arosa Y., de la Fuente R. (2020). Refractive Index Spectroscopy and Material Dispersion in Fused Silica Glass. Opt. Lett..

[50] Shukla V., Bajpai M., Singh D.K., Singh M., Shukla R. (2004). Review of Basic Chemistry of UV-curing Technology. Pigment Resin Technol..

[51] Altun-Çiftçioğlu G.A., Ersoy-Meriçboyu A., Henderson C.L. (2011). Stochastic Modeling and Simulation of Photopolymerization Process. Polym. Eng. Sci..

[52] Mondal D., Willett T.L. (2022). Enhanced Mechanical Performance of mSLA-Printed Biopolymer Nanocomposites Due to Phase Functionalization. J. Mech. Behav. Biomed. Mater..

[53] Bischoff F. (1972). Organic Polymer Biocompatibility and Toxicology. Clin. Chem..

[54] Zhang W., Li Y., Niu J., Chen Y. (2013). Photogeneration of Reactive Oxygen Species on Uncoated Silver, Gold, Nickel, and Silicon Nanoparticles and Their Antibacterial Effects. Langmuir.

[55] Hassanpour M., Narongdej P., Alterman N., Moghtadernejad S., Barjasteh E. (2024). Effects of Post-Processing Parameters on 3D-Printed Dental Appliances: A Review. Polymers.

[56] El-Gohary R.M., El-Shafai N.M., El-Mehasseb I.M., Ghamry H.I., Alshahrani M.Y., Beltagi A.M. (2025). Design Plasmonic Nanostructure of Silicon Dioxide and Titanium Dioxide Loaded on a Nano Surface for Clean Water Production through Photocatalysis and Electrochemical Techniques. Mater. Res. Bull..

[57] Lehman S.E., Morris A.S., Mueller P.S., Salem A.K., Grassian V.H., Larsen S.C. (2016). Silica Nanoparticle-Generated ROS as a Predictor of Cellular Toxicity: Mechanistic Insights and Safety by Design. Environ. Sci. Nano.

[58] Gong C., Tao G., Yang L., Liu J., He H., Zhuang Z. (2011). The Role of Reactive Oxygen Species in Silicon Dioxide Nanoparticle-Induced Cytotoxicity and DNA Damage in HaCaT Cells. Mol. Biol. Rep..

[59] David Gara P.M., Garabano N.I., Llansola Portoles M.J., Moreno M.S., Dodat D., Casas O.R., Gonzalez M.C., Kotler M.L. (2012). ROS Enhancement by Silicon Nanoparticles in X-Ray Irradiated Aqueous Suspensions and in Glioma C6 Cells. J. Nanopart. Res..

[60] Faisal M., Faizan M., Soysal S., Alatar A.A. (2024). Synergistic Application of Melatonin and Silicon Oxide Nanoparticles Modulates Reactive Oxygen Species Generation and the Antioxidant Defense System: A Strategy for Cadmium Tolerance in Rice. Front. Plant Sci..

[61] Hu H., Fan X., Guo Q., Wei X., Yang D., Zhang B., Liu J., Wu Q., Oh Y., Feng Y. (2019). Silicon Dioxide Nanoparticles Induce Insulin Resistance through Endoplasmic Reticulum Stress and Generation of Reactive Oxygen Species. Part. Fibre Toxicol..

[62] Golenkina E., Viryasova G., Galkina S., Gaponova T., Sud’ina G., Sokolov A. (2018). Fine Regulation of Neutrophil Oxidative Status and Apoptosis by Ceruloplasmin and Its Derivatives. Cells.

[63] Kostyuk S.V., Konkova M.S., Ershova E.S., Alekseeva A.J., Smirnova T.D., Stukalov S.V., Kozhina E.A., Shilova N.V., Zolotukhina T.V., Markova Z.G. (2013). An Exposure to the Oxidized DNA Enhances Both Instability of Genome and Survival in Cancer Cells. PLoS ONE.

[64] Sharapov M.G., Novoselov V.I., Fesenko E.E., Bruskov V.I., Gudkov S.V. (2017). The Role of Peroxiredoxin 6 in Neutralization of X-Ray Mediated Oxidative Stress: Effects on Gene Expression, Preservation of Radiosensitive Tissues and Postradiation Survival of Animals. Free. Radic. Res..

[65] Kurashova N.A., Dashiev B.G., Kolesnikov S.I., Kolesnikova L.I. (2021). Indicators of the Lipid Peroxidation—Antioxidant Protection System as Important Metabolic Markers of Reproductive Potential in Men. Bull. Exp. Biol. Med..

[66] Gudkov S.V., Gudkova O.Y., Chernikov A.V., Bruskov V.I. (2009). Protection of Mice against X-Ray Injuries by the Post-Irradiation Administration of Guanosine and Inosine. Int. J. Radiat. Biol..

[67] Krikunova L.I., Mkrtchian L.S., Zamulaeva I.A., Yakimova A.O., Dzikovskaya L.A., Degtiareva E.S., Khailova Z.V., Ivanov S.A., Kaprin A.D. (2025). Correction of Oxidative Stress with Glutathione-Based Agents in Women with Hyperproliferative Diseases Living in Radiation-Contaminated Areas: A Prospective Study. Gynecology.

[68] Shtarkman I.N., Gudkov S.V., Chernikov A.V., Bruskov V.I. (2008). Effect of Amino Acids on X-Ray-Induced Hydrogen Peroxide and Hydroxyl Radical Formation in Water and 8-Oxoguanine in DNA. Biochemistry.

[69] Ivanov V.E., Usacheva A.M., Chernikov A.V., Bruskov V.I., Gudkov S.V. (2017). Formation of Long-Lived Reactive Species of Blood Serum Proteins Induced by Low-Intensity Irradiation of Helium-Neon Laser and Their Involvement in the Generation of Reactive Oxygen Species. J. Photochem. Photobiol. B Biol..

[70] Gudkov S.V., Shtarkman I.N., Chernikov A.V., Usacheva A.M., Bruskov V.I. (2007). Guanosine and Inosine (Riboxin) Eliminate the Long-Lived Protein Radicals Induced X-Ray Radiation. Dokl. Biochem. Biophys..

[71] Davies M. (2014). Long-Lived Reactive Species Formed on Proteins Induce Changes in Protein and Lipid Turnover. Free Radic. Biol. Med..

[72] Belov S.V., Danyleiko Y.K., Glinushkin A.P., Kalinitchenko V.P., Egorov A.V., Sidorov V.A., Konchekov E.M., Gudkov S.V., Dorokhov A.S., Lobachevsky Y.P. (2021). An Activated Potassium Phosphate Fertilizer Solution for Stimulating the Growth of Agricultural Plants. Front. Phys..

[73] Besinis A., De Peralta T., Handy R.D. (2012). The Antibacterial Effects of Silver, Titanium Dioxide and Silica Dioxide Nanoparticles Compared to the Dental Disinfectant Chlorhexidine on Streptococcus Mutans Using a Suite of Bioassays. Nanotoxicology.

[74] Williams F.E., Lee A.K., Orandi S., Lewis D.M. (2018). Antibacterial Action of Functional Silicon Dioxide: An Investigation of the Attachment and Separation of Bacteria. Environ. Technol..

[75] Lozins R., Selga T., Ozoliņš D. (2020). Microorganism Adhesion Using Silicon Dioxide: An Experimental Study. Heliyon.

[76] Alavi M., Thomas S., Sreedharan M. (2022). Modification of Silica Nanoparticles for Antibacterial Activities: Mechanism of Action. Micro Nano Bio Asp..

[77] Wang Z., Zhai X., Sun Y., Yin C., Yang E., Wang W., Sun D. (2020). Antibacterial Activity of Chlorogenic Acid-Loaded SiO_2_ Nanoparticles Caused by Accumulation of Reactive Oxygen Species. Nanotechnology.

[78] Barmina E.V., Gudkov S.V., Simakin A.V., Shafeev G.A. (2017). Stable Products of Laser-Induced Breakdown of Aqueous Colloidal Solutions of Nanoparticles. J. Laser Micro Nanoeng..

[79] Sevost’yanov M.A., Nasakina E.O., Baikin A.S., Sergienko K.V., Konushkin S.V., Kaplan M.A., Seregin A.V., Leonov A.V., Kozlov V.A., Shkirin A.V. (2018). Biocompatibility of New Materials Based on Nano-Structured Nitinol with Titanium and Tantalum Composite Surface Layers: Experimental Analysis in Vitro and in Vivo. J. Mater. Sci. Mater. Med..

[80] Shiloach J., Fass R. (2005). Growing *E. coli* to high cell density—A historical perspective on method development. Biotechnol. Adv..

[81] Boi P., Manti A., Pianetti A., Sabatini L., Sisti D., Rocchi M.B., Bruscolini F., Galluzzi L., Papa S. (2015). Evaluation of *Escherichia coli* Viability by Flow Cytometry: A Method for Determining Bacterial Responses to Antibiotic Exposure. Cytom. Part B.

[82] Sevostyanov M.A., Kolmakov A.G., Sergiyenko K.V., Kaplan M.A., Baikin A.S., Gudkov S.V. (2020). Mechanical, Physical–Chemical and Biological Properties of the New Ti–30Nb–13Ta–5Zr Alloy. J. Mater. Sci..

